# Ultrafast nano-oscillators based on interlayer-bridged carbon nanoscrolls

**DOI:** 10.1186/1556-276X-6-470

**Published:** 2011-07-25

**Authors:** Zhao Zhang, Teng Li

**Affiliations:** 1 Department of Mechanical Engineering, University of Maryland, College Park, MD 20742, USA; 2 Maryland NanoCenter, University of Maryland, College Park, MD 20742, USA

**Keywords:** carbon nanoscroll, graphene, carbon nanotube, nano-oscillator, molecular dynamics

## Abstract

We demonstrate a viable approach to fabricating ultrafast axial nano-oscillators based on carbon nanoscrolls (CNSs) using molecular dynamics simulations. Initiated by a single-walled carbon nanotube (CNT), a monolayer graphene can continuously scroll into a CNS with the CNT housed inside. The CNT inside the CNS can oscillate along axial direction at a natural frequency of tens of gigahertz. We demonstrate an effective strategy to reduce the dissipation of the CNS-based nano-oscillator by covalently bridging the carbon layers in the CNS. We further demonstrate that such a CNS-based nano-oscillator can be excited and driven by an external AC electric field, and oscillate at more than 100 GHz. The CNS-based nano-oscillators not only offer a feasible pathway toward ultrafast nano-devices but also hold promise to enable nanoscale energy transduction, harnessing, and storage (e.g., from electric to mechanical).

## Introduction

Significant research progress on graphene in past several years has enabled the exploration of carbon nanoscrolls (CNSs) [1-3], a one-dimensional carbon nanomaterial that is distinct from carbon nanotubes (CNTs). A CNS is formed by rolling up a monolayer graphene into a spiral multilayer nanostructure, whose core size is highly tunable by relative sliding between adjacent layers [4,5]. In other words, a CNS is topologically open, fundamentally distinct from a tubular CNT, which is topologically closed (e.g., whose core size can only be changed slightly by stretching the carbon-carbon (C-C) bonds). The open and highly tunable structure of CNSs, combining with the exceptional mechanical and electronic properties inherited from the basal graphene [6-9], has inspired an array of novel nano-device applications, such as hydrogen storage medium [10,11], water and ion channels [12], radially breathing nano-oscillators [13], and translational nano-actuators [14]. In this paper, we demonstrate ultrafast CNS-based axial nano-oscillators that operate at frequencies from tens of gigahertz to more than 100 GHz, using molecular dynamic (MD) simulations.

**Figure 1:**
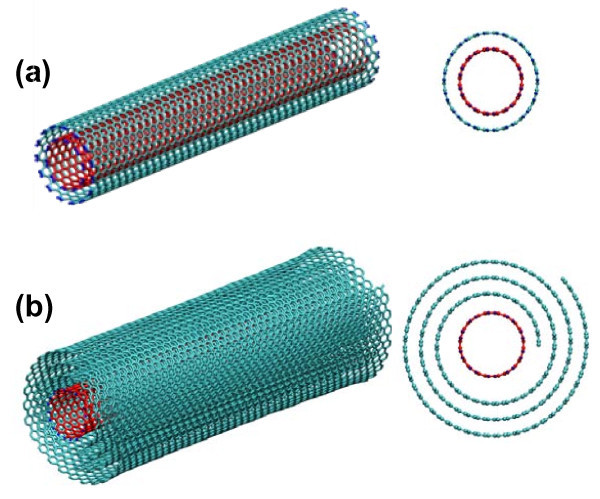
**(a) a DWCNT and (b) a CNS with a SWCNT housed inside**. Perspective view (left) and end view (right). When displaced from its equilibrium position along axial direction, the inner tube (red) can oscillate inside the outer tube (cyan) or CNS (cyan), at gigahertz of frequency.

Axial nano-oscillators based on multi-walled CNTs (MWCNTs) have been proposed previously [15]. In the proposed MWCNT-based axial nano-oscillator, the ends of the outer tubes of a MWCNT are opened. When the inner tubes are displaced from their original position along the axial direction and then released, the restoring force from the outer tubes pulls the inner ones back. Due to the ultralow friction between the carbon layers, the inner tubes can oscillate along its axial direction, and the natural frequency of the oscillation is estimated to be on the order of gigahertz [15-17]. Figure 1a illustrates a double-walled CNT (DWCNT)-based axial nano-oscillator. Enthusiasm for MWCNT-based axial nano-oscillators aside, the realization of such promising nano-devices hinges upon feasible fabrication techniques. For example, well-controlled opening of the ends of the outer tubes of a MWCNT and chemical treatment of the inner tubes in a MWCNT (e.g., doping or polarization) still remain as significant challenges. As a result, successful fabrication of MWCNT-based axial nano-oscillators has not yet been demonstrated, let alone the exploration of exciting the axial oscillation of such nano-oscillators via external interferences [18-20].

It has been recently demonstrated that a CNT of suitable diameter can initiate the scrolling of a monolayer graphene on a substrate into a CNS [21]. The CNT near the edge of the graphene can help overcome the initial energy barrier for the scrolling of graphene. Once the scrolling is initiated, the graphene can spontaneously roll up into a CNS. The resulting CNS/CNT nanostructure has the two ends of the CNS naturally open and a CNT housed inside the CNS (e.g., Figure 1b). Similar scrolling of a graphene oxide layer initiated by a MWCNT has been experimentally demonstrated recently [22]. As to be detailed later, when the CNT is displaced partially out of the CNS along the axial direction, the van der Waals force acting on the two ends of the CNT is not balanced and the resultant force on the CNT serves as the restoring force to pull the CNT back into the CNS. Given the ultralow CNT-CNS friction similar to the inter-tube friction in a MWCNT, the CNT in the CNS is shown to be able to oscillate at a frequency of tens of gigahertz. In this paper, we use molecular dynamics simulations to perform systematic investigation of the characteristics of the ultrafast oscillation of the abovementioned CNS-based axial nano-oscillators. We propose a feasible strategy to significantly reduce the energy dissipation of the CNS-based nano-oscillators. We further demonstrate that the CNS-based nano-oscillators can be excited by an external AC electrical field and oscillate at a frequency more than 100 GHz. A distinct advantage of the CNS-based nano-oscillators against the MWCNT-based ones is as follows. The CNT and the basal graphene are fabricated separately before the scrolling process. For example, the CNT and the graphene can be treated differently and thus possess different features, such as defects, chirality, and polarization. These features make it possible to significantly enhance the performance of the CNS-based axial nano-oscillators, as to be detailed later in this paper. With the ever maturing fabrication technique of high-quality graphene, CNS-based axial nano-oscillators hold promise to become a viable approach to achieving nanoscale gigahertz mechanical oscillators. In particular, the excitation of CNS-based nano-oscillators under external interferences demonstrates their great potential as nanoelectromechanical systems (NEMS) for nanoscale energy transduction (e.g., from electrical and/or magnetic to mechanical), harvesting, and storage (e.g., as mechanical oscillation).

## Results and discussions

### CNT-initiated scrolling of graphene into a CNS

**Figure 2:**
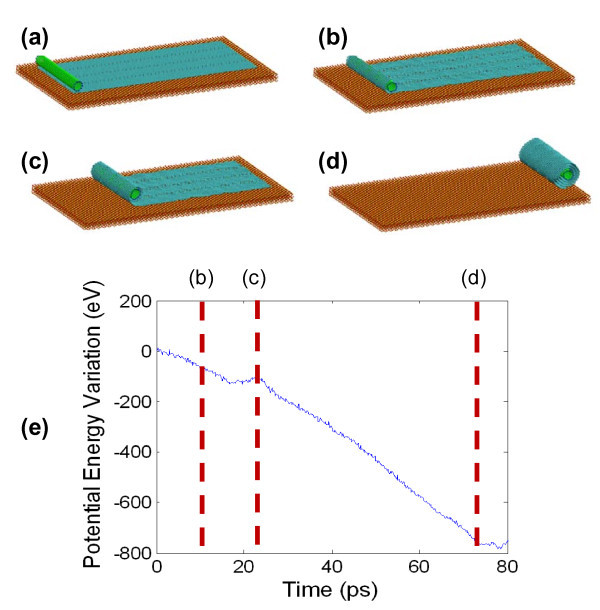
**CNT-initiated scrolling of graphene into a CNS**. (**a-d**) Snapshots of the graphene scrolling into a CNS initiated by a (10, 10) SWCNT, before equilibration, at 10, 22, and 76 ps, respectively. (**e**) The variation in the total potential energy of the system as a function of simulation time.

The CNS-based axial nano-oscillator depicted in Figure 1b was formed using a 10-nm-long (10, 10) single-walled CNT (SWCNT) to initiate the scrolling of a 10 nm by 30 nm graphene along its long (armchair) edge. The formation of the CNS/CNT nanostructure is similar to that described in Ref. [21]. As shown in Figure 2a, the graphene is supported by a SiO_2 _substrate, with a (10, 10) single wall CNT placed along the left edge of the graphene. The substrate is 34 nm long, 14 nm wide, and 1 nm thick. In the MD simulations, the C-C bonds in the CNT and CNS are described by the second-generation Brenner potential [23], which allows for C-C covalent bond forming and breaking. The non-bonded C-C interaction is described by a Lennard-Jones pair potential [24]. The graphene-substrate interaction is considered in the same way as in Ref. [21]. The MD simulations are carried out using LAMMPS [25] with canonical ensemble at 500 K and with time step of 1 fs.

Initiated by the CNT, the graphene first separates from the substrate and curls up to wrap the CNT (Figure 2b). Once the overlap between the left edge and the flat portion of the graphene forms (Figure 2c), graphene starts to scroll continuously into a CNS (Figure 2d) with the CNT housed inside. An additional movie file shows the CNT-initiated scrolling of graphene into a CNS (see Additional file 1). Figure 2e shows the decrease of the total potential energy due to the graphene wrapping the CNT and further scrolling into a CNS. Our simulations show that there is no appreciable difference between the scrolling of a pristine graphene (without any defects) and that of a graphene with defects. For example, the graphene shown in Figure 2 has patterned vacancies along three parallel lines, the effect of which is to be detailed later.

### Oscillation of a CNT housed inside a naturally formed CNS

**Figure 3:**
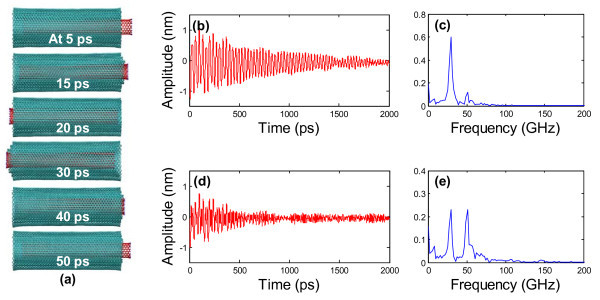
**Oscillation of a CNT housed inside a naturally formed CNS**. (**a**) Snapshots of the axial oscillation of the CNS-based nano-oscillator at 5, 15, 20, 30, 40, and 50 ps, respectively. Note the coupled axial oscillations of the CNT and the CNS itself. The evolution of (**b**) the absolute amplitude and (**d**) the relative amplitude of the CNT oscillation inside the CNS, as a function of simulation time, respectively. The FFT analysis of the absolute amplitude (**c**) and the relative amplitude (**e**) for the first 500 ps reveals a frequency of the oscillation of the CNT inside the CNS (29.4 GHz) and that of the oscillation of the CNS itself (50.9 GHz), respectively. The simulations are carried out at 100 K.

The CNS-based nano-oscillator formed by scrolling up a pristine graphene is first equilibrated for 50 ps at 100 K, and then, the CNT housed inside is assigned a velocity 2.5 Å/ps along its axial direction to initiate the oscillation. In order to constrain the rigid body motion of the nano-oscillator, two rows of carbon atoms along the axial direction on the outermost shell of the CNS are fixed. Figure 3a shows the snapshots of the axial oscillation of the CNS/CNT nanostructure at 5, 15, 20, 30, 40, and 50 ps, respectively (see Additional file 2 for a video of the oscillation). The simulations are carried out at 100 K. The axial motion of the CNT is excluded in the calculation of temperature. Besides the oscillation of the CNT inside the CNS, the CNS itself also oscillates through interlayer relative sliding in axial direction, initiated by the reaction force from the CNT (i.e., opposite to the restoring force applied on the CNT). The reaction force pulls the inner shells of the CNS to slide outward during the CNT oscillation; thus, the CNS itself starts to oscillate accompanying the CNT motion. As a result, the oscillation of the CNS/CNT nanostructure is indeed the coupled CNT oscillation and that of the CNS itself. It needs to be pointed out that the CNS self-oscillation is not only driven by the van der Waals-type reaction force between the CNS and the CNT but also affected by the in-plane shear rigidity of the basal graphene. The different energetic interplays for the CNT oscillation and the CNS self-oscillation lead to a rather irregular coupled oscillation, similar to the axial oscillation observed in a MWCNT [26].

To further decipher the coupled oscillation of the CNS/CNT nanostructure, we define two oscillation amplitudes: the absolute amplitude which is the axial distance from the left end of the CNT to the outermost atom at the left end of the CNS (which is fixed) and the relative amplitude which is the axial distance from the left end of the CNT to the innermost atom at the left end of the CNS (which moves as the CNS oscillates). Figure 3b, d plot the absolute and relative amplitudes as the function of simulation time, respectively. While the absolute amplitude captures the oscillation of the CNT, the relative amplitude characterizes the coupled oscillation of the CNS/CNT nanostructure, which is more irregular and decays faster. Fast Fourier Transform (FFT) analysis is also performed for the first 500 ps of the oscillation. FFT of the absolute amplitude shows a peak at 29.4 GHz (Figure 3c), which represents the frequency of CNT oscillation. By contrast, FFT of the relative amplitude shows two peaks, 29.4 and 50.9 GHz, respectively (Figure 3e). While the first peak corresponds to the frequency of CNT oscillation inside the CNS, the second peak reveals the frequency of the CNS self-oscillation. The higher frequency of the CNS self-oscillation results from the restoring force contributed by both the non-bonded van der Waals force among carbon layers and the covalent C-C bonding force in the basal graphene.

### Oscillation of a CNT housed inside an interlayer-bridged CNS

While the ultrafast oscillation of the CNT inside the CNS at tens of gigahertz is encouraging, the quick dissipation and rather irregular behavior of the oscillation definitely limit the potential application of CNS-based nano-oscillators as NEMS devices. The quick dissipation and irregular oscillation result from the coupled oscillation, during which the kinetic energy of the CNT is continuously transduced into the self-oscillation of the CNS and then dissipates by the friction due to interlayer sliding. To address this issue, we next demonstrate a feasible and effective strategy to suppress the relative interlayer sliding in the CNS, which can lead to a much more sustainable ultrafast CNS-based nano-oscillator.

Both simulations and experiments have shown that when a MWCNT is treated by ion irradiation, some carbon atoms can be knocked off, leaving vacancies in the tubes of the MWCNT [27]. Upon heating, the carbon atoms near the vacancies tend to form covalent bonds with other similar carbon atoms in a neighboring tube, driven by the reduction of high-energy dangling bonds of these carbon atoms. As a result, the tubes in the MWCNT are covalently bridged, leading to a significant increase of the inter-tube shear rigidity of the MWCNT. In other words, the relative inter-tube sliding in such a bridged MWCNT involves breaking the covalent C-C bridging bonds, thus is energetically unfavorable. The ion irradiation induced vacancies are also used to facilitate the bridging bond formation among SWCNTs to form CNT bundles [28,29]. Inspired by these previous studies, next we demonstrate that vacancies can facilitate the formation of interlayer bridging bonds in a CNS, which in turn can effectively suppress the interlayer relative sliding in the CNS.

**Figure 4:**
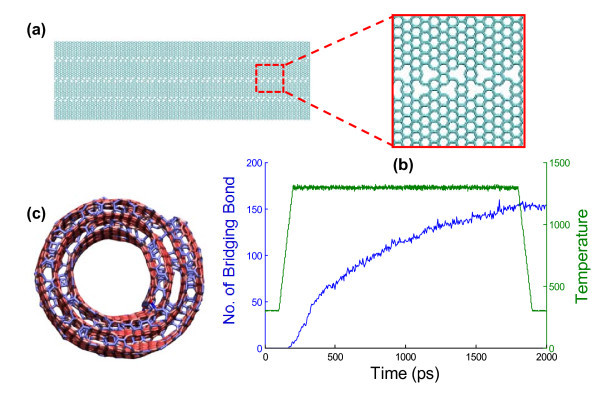
**The formation of interlayer bridging bonds in a CNS**. (**a**) The graphene with patterned vacancies. (**b**) The evolution of the number of interlayer bridging bond in the CNS and the temperature change as a function of time, respectively. Note that the bridging bonds remain after cooling down to room temperature. (**c**) The end view of the interlayer-bridged CNS after the heat treatment. The color shades represent potential energy level of the carbon atoms. Here, the SWCNT housed inside the CNS is not shown for visual clarity.

Instead of using a pristine graphene, we use graphene with patterned vacancies to form a CNS. The vacancies in the graphene are patterned along three parallel lines in the scrolling direction (Figure 4a) to facilitate bridging bond formation after scrolling. In reality, such vacancies can be introduced using focus ion beam to irradiate the graphene along those parallel lines. A SWCNT is used to initiate the scrolling of the aforementioned graphene with patterned vacancies. The carbon atoms at the two ends of the SWCNT are saturated by hydrogen atoms so that no bridging bonds can be formed between the SWCNT and the CNS. After the scrolling process of the basal graphene with vacancies, the resulting CNS/CNT nanostructure is first heated up from 300 to 1,300 K in 100 ps, then maintained at 1,300 K for 1,600 ps, and finally cooled down back to 300 K in 100 ps. As shown in Figure 4b, interlayer bridging bonds start to form after the temperature reaches 1,000 K. The total number of interlayer bridging bonds in the CNS increases as the temperature further increases to and maintains at 1,300 K, and gradually saturates (see Additional file 3 for a video of the dynamic process of interlayer bridging bond formation). After cooled down to room temperature, the interlayer bridging bonds formed at high temperature remain in the CNS. Figure 4c depicts the end view of the bridged CNS after the heat treatment. Besides the interlayer bridging bonds inside the CNS, bridging bonds are also formed along the unsaturated edges of the CNS (i.e., at the two ends of the CNS and the two edges along its axial direction). No bridging bond is formed between the CNS and the SWCNT with saturated ends.

**Figure 5:**
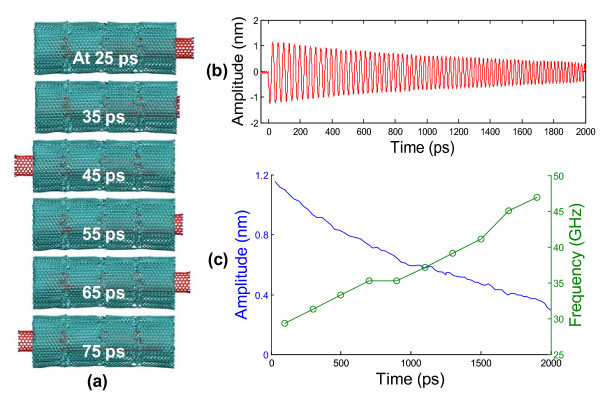
**Oscillation of a CNT housed inside an interlayer-bridged CNS**. (**a**) Snapshots of the axial oscillation of the bridged-CNS-based nano-oscillator at 25, 35, 45, 55, 65, and 75 ps, respectively. Note the oscillation of the CNS itself is fully constrained by the interlayer bridging bonds. (**b**) The evolution of CNT oscillation amplitude. (**c**) The peak amplitude of each oscillation cycle and the corresponding oscillation frequency as a function of time, respectively. The simulations are carried out at 100 K.

The oscillation of the SWCNT housed inside the interlayer-bridged CNS is then investigated following the similar procedure used for that of the SWCNT inside the un-bridged CNS. Figure 5a shows the snapshots of the axial oscillation of the interlayer-bridged CNS/CNT nanostructure at 25, 35, 45, 55, 65, and 75 ps, respectively (see Additional file 4 for a video of the oscillation). No appreciable relative sliding among the CNS layers is found during the oscillation of the CNT. In other words, the self-oscillation of the CNS is effectively suppressed by the interlayer bridging bonds. This is further confirmed by the negligible difference between the absolute amplitude and relative amplitude of the CNT as defined above. Figures 5b plots the absolute amplitude of CNT as a function of simulation time. Compared with the oscillation of the CNT inside an un-bridged CNS, the CNT oscillation inside an interlayer-bridged CNS is much more regular. Also evident in Figure 5b is the slower decay of the oscillation amplitude when compared with Figure 3b, which results from the suppression of energy dissipation due to interlayer relative sliding in the CNS. Figure 5c plots the peak amplitude of each oscillation cycle and the corresponding oscillation frequency obtained from FFT analysis as a function of simulation time, respectively. The initial frequency of the CNT oscillation is 29.4 GHz when the oscillation amplitude is about 1.15 nm, and the oscillation frequency at 2 ns is 47.0 GHz when the oscillation amplitude is about 0.30 nm. The oscillation frequency increases monotonically as the oscillation amplitude decreases over the time. Such a dependence of oscillation frequency on oscillation amplitude is consistent with the MWCNT-based axial oscillators as reported in earlier studies [30,31].

**Figure 6:**
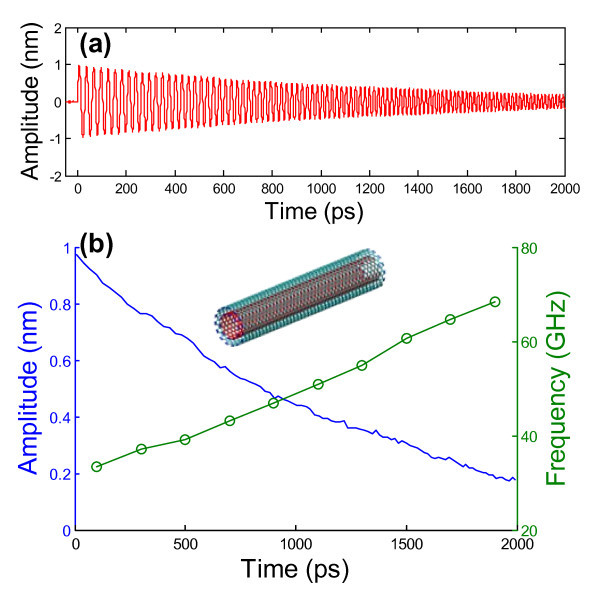
**Oscillation of a DWCNT-based nano-oscillator**. (**a**) The evolution of the oscillation amplitude of the inner tube of a (10, 10)/(15, 15) DWCNT. (**b**) The peak oscillation amplitude of each cycle and the corresponding oscillation frequency as a function of time, respectively. The simulations are carried out at 100 K.

We next compare the performance of bridged-CNS-based nano-oscillators with that of MWCNT-based nano-oscillators. Our studies show that there is negligible difference in the oscillation behaviors between an MWCNT-based nano-oscillator and a DWCNT-based one if only the innermost tube oscillates and the DWCNT is identical to the two innermost tubes of the MWCNT. Thus, here we report the simulation results of the oscillation behaviors of a (10, 10)/(15, 15) DWCNT, following the similar procedure aforementioned. In order to constrain the rigid body motion of the nano-oscillator, one ring of carbon atoms in the middle of the outer tube of the DWCNT are fixed. The inner tube is assigned a velocity of 2.5 Å/ps along its axial direction to initiate the oscillation. The oscillation amplitude, defined as the axial distance from the left end of the inner tube to the left end of the outer tube, is plotted as a function of simulation time in Figure 6a. The peak oscillation amplitude of each cycle and the corresponding oscillation frequency as a function of time are shown in Figure 6b. While the initial velocity of the inner tube is the same, the resulting initial oscillation amplitude of the DWCNT-based nano-oscillator is slightly smaller than that of the bridged-CNS-based nano-oscillator. Such a difference results from the slight difference in the geometry between the outer tube of the DWCNT (a perfect tube) and the innermost layer of the bridged-CNS (a tube that is cut in axial direction and then slightly displaced radially), leading to a restoring force of the DWCNT-based nano-oscillator modestly larger than that of the bridged-CNS-based one. The difference in the restoring force also explains the relatively higher oscillation frequency of the DWCNT-based nano-oscillator than that of the bridged-CNS-based one for a given oscillation magnitude. Nonetheless, the comparison between Figures 5c and 6b shows that the bridged-CNS-based nano-oscillator has a modestly slower dissipation rate than the DWCNT-based nano-oscillator. For example, it takes about 1,000 ps for the magnitude of DWCNT-based nano-oscillator to decay from 0.9 to 0.4 nm, while it takes 1,300 ps for the bridged-CNS-based nano-oscillator. We also estimate the quality factor of a nano-oscillator from the evolution of its oscillation amplitude (e.g., Figures 5b and 6a) to be , where *N *is the total number of oscillation cycles in the MD simulation and *A_i _*denotes the peak amplitude of the *i*th cycle. For the bridged-CNS-based nano-oscillator (Figure 5), *Q *≈ 207, and for the DWCNT-based nano-oscillator (Figure 6), *Q *≈ 192. Such a comparison of the oscillator performance agrees with the above comparison based on the damping time for a given oscillation amplitude decay. Earlier studies have shown that the translational energy in a DWCNT-based oscillator is mainly dissipated via a wavy deformation in the outer tube undergoing radial vibration [32]. In a bridged CNS, the constraint from the covalent interlayer bridging bonds can largely suppress the radial deformation of all layers in the CNS. In other words, the bridged CNS serves as a thick-walled tubular nanostructure with a much higher rigidity in both axial and radial directions than a MWCNT. As a result, the axial oscillation of the SWCNT housed inside the bridged CNS is more sustainable than that inside a MWCNT.

### Effects of temperature and commensuration on the nano-oscillator performance

**Figure 7:**
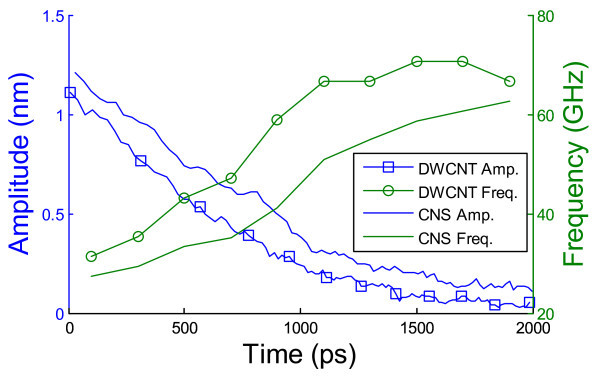
**The comparison between the bridged-CNS-based nano-oscillator and the DWCNT-based nano-oscillator at 300 K**.

To understand the effect of temperature on the performance of the bridged-CNS-based nano-oscillator, Figure 7 compares the peak oscillation amplitude of each cycle and the corresponding oscillation frequency as a function of time for a bridged-CNS-based nano-oscillator and a DWCNT-based nano-oscillator at 300 K. For both nano-oscillators, the decay of the oscillation magnitude at 300 K is modestly faster than that at 100 K, while the corresponding oscillation frequency is slightly higher than that at 100 K. At higher temperature, the thermal fluctuation of the carbon atoms in the nano-oscillators becomes more energetic, resulting in rougher surfaces of both the oscillating CNT and the carbon layers of the housing CNT or CNS and therefore increased interlayer friction. Nonetheless, the bridged-CNS-based nano-oscillators still have a modestly slower dissipation rate than the DWCNT-based nano-oscillator at an elevated temperature.

**Figure 8:**
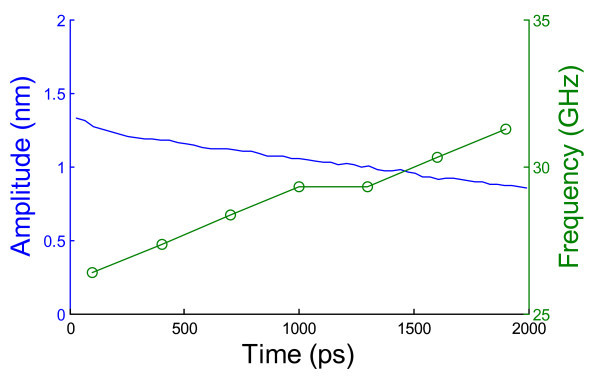
**Oscillation of an incommensurate bridged-CNS-based nano-oscillator**. The peak oscillation amplitude of each cycle and the corresponding oscillation frequency as a function of time for a (15, 0) SWCNT inside the interlayer-bridged CNS, respectively. The simulations are carried out at 100 K.

Besides the temperature, the commensuration between the oscillating CNT and the housing CNT or CNS also influences the oscillation performance. It has been shown that the DWCNT-based oscillators with incommensurate inner and outer tubes have lower inter-tube friction force than the commensurate ones, leading to a much slower dissipation rate [30,33]. To demonstrate the similar effect in bridged-CNS-based nano-oscillators, we replace the (10, 10) SWCNT that is housed inside and commensurate with the interlayer-bridged CNS with an incommensurate (15, 0) SWCNT (whose diameter is very close to (10, 10) SWCNT). Figure 8 reveals that the dissipation rate of the incommensurate bridged-CNS-based nano-oscillator (approximately 0.237 nm/ns) is much slower than that of the commensurate one (approximately 0.429 nm/ns). These results demonstrate an effective strategy to further enhance the performance of bridged-CNS-based nano-oscillators using an incommensurate oscillating SWCNT inside. Our further studies show that the CNT-initiated scrolling of graphene is insensitive to the chirality of the CNT and the basal graphene. This further validates the feasibility of such a strategy since the CNT and the basal graphene can be first synthesized and selected separately and then assembled. By contrast, synthesizing MWCNTs with controlled commensuration among constituent tubes still remains as a grand challenge, let alone leveraging such a strategy to improve the performance of MWCNT-based nano-oscillators.

### Oscillation of the CNS/CNT nano-oscillator excited and driven by an external electric field

**Figure 9:**
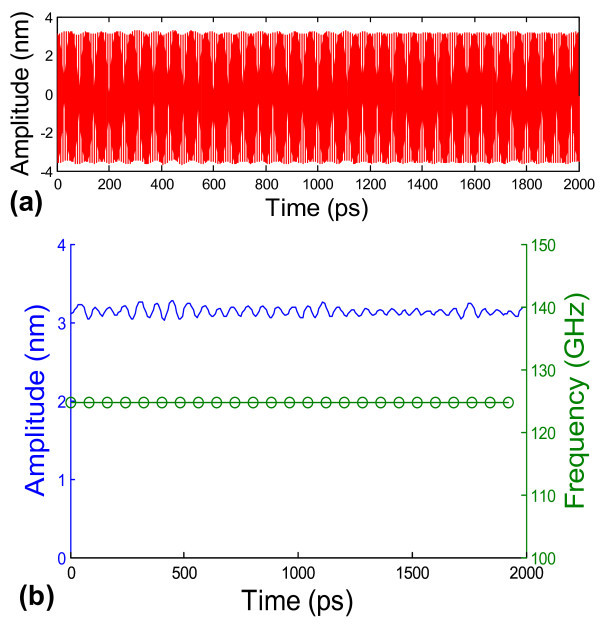
**Oscillation of the CNS/CNT nano-oscillator excited and driven by an external electric field**. (**a**) The oscillation of the CNT for an external electrical field with an ac frequency of 125 GHz. (**b**) The peak oscillation amplitude of each cycle and the corresponding oscillation frequency as a function of time. The external AC electrical field can override the natural frequency of the CNS-based nano-oscillator. There is no appreciable decay of peak oscillation amplitude. The simulations are carried out at 100 K.

We further demonstrate that the bridged-CNS-based nano-oscillators can be excited and driven by an external electric field, a crucial feature to enable their potential application in ultrafast NEMS devices. For the MWCNT-based nano-oscillators, it has been proposed that by inducing net charge [20] or electric dipole [18] into the inner tube, the carbon atoms in the charged/polarized inner tube are subjected to electrostatic capacitive force in an external electric field, which could be potentially used to initialize the oscillation. Controlled charging/polarization of the inner tube of an MWCNT requires manipulation with sub-nanometer precision, thus remains rather challenging to achieve experimentally. However, such a strategy can become feasible for bridged-CNS-based nano-oscillators. For example, the SWCNT to be housed inside the interlayer-bridged CNS can be treated to possess net charges or dipoles before used to initiate the scrolling of the basal graphene that remains electrically neutral. Subject to an external AC electric field, the oscillation of the SWCNT housed inside the interlayer-bridged CNS can be initiated and driven by the alternating capacitive force. As a benchmark of such a strategy, Figure 9 shows the oscillation of the bridged-CNS-based nano-oscillator excited and then driven by a square-wave AC electric field with a frequency of 125 GHz. The amplitude of the resulting capacitive force acting on the SWCNT is 0.02 eV/Å per atom. Because such a driving force is much larger than the intensity of the intrinsic van der Waals restoring force between the atoms in the SWCNT and the CNS (approximately 0.0004 eV/Å per atom), the oscillation driven by the external electric field can override the natural oscillation of the bridged-CNS-based nano-oscillator. The slightly asymmetric oscillation amplitude profile in Figure 9a (e.g., offset by about 0.2 nm) may possibly result from the slightly biased restoring force by the non-uniform atomic structure of the bridged CNS (e.g., due to randomly distributed interlayer bridging bonds). Figure 9b shows that the frequency of the resulting oscillation is identical to that of the external AC electric field. Furthermore, there is no appreciable decay in the oscillation amplitude, whose peak value in each oscillation cycle only fluctuates within 5%. In other words, the oscillation driven by the external electric field is highly sustainable. Our further studies show that the resulting oscillation of the bridged-CNS-based nano-oscillator can be further fine-tuned in a certain range under an external AC electric field of suitable frequency and magnitude. These explorations further demonstrate the potential to leverage CNS-based nano-oscillators to convert the electric energy of an external AC field into mechanical energy in the form of ultrafast oscillation. With proper treatment of the oscillating CNT, the above strategy can be potentially adapted to transduce and harvest electromagnetic and thermal energy into ultrafast mechanical oscillation [16,34].

## Conclusions

To conclude, we demonstrate a new type of ultrafast axial nano-oscillators based on CNS. Such a nano-oscillator consists of a SWCNT that is housed inside a CNS, which can be feasibly formed by the SWCNT-initiated scrolling of a basal monolayer graphene. The unique topological structure of the CNS-based nano-oscillator offers a viable pathway to fabricating ultrafast axial nano-oscillators, addressing a significant challenge that still remains for the previously proposed MWCNT-based axial nano-oscillator. We propose an effective and feasible strategy to reduce the oscillation dissipation of the CNS-based nano-oscillators by introducing interlayer bridging bonds in the CNS. The performance of the resulting bridged-CNS-based nano-oscillators is comparable or modestly better than the MWCNT-based ones. We further demonstrate the highly sustainable oscillation of the bridged-CNS-based nano-oscillators that can be excited and driven by an external AC electric field. With the ever maturing fabrication of high-quality monolayer graphene and nanofabrication technique of patterning nanoscale building blocks, we envision a novel approach to harnessing and storing energy at nanoscale and over large area, enabled by distributing CNS-based nano-oscillators on an electronic surface.

## Abbreviations

CNT: carbon nanotube; CNS: carbon nanoscroll: SWCNT: single-walled carbon nanotube; DWCNT: double-walled carbon nanotube; MWCNT: multi-walled carbon nanotube; MD: molecular dynamic.

## Competing interests

The authors declare that they have no competing interests.

## Authors' contributions

TL designed and supervised the research, ZZ carried out simulations, TL and ZZ analyzed the data, and TL and ZZ wrote the paper.

## Supplementary Material

Additional file 1 **CNS formation**. A video showing the CNT-initiated scrolling of graphene into a CNS. Click here for file

Additional file 2 **Unbridged CNS**. A video showing the oscillation of a CNT housed inside a naturally formed CNS. Click here for file

Additional file 3 **Bridging bond formation**. A video showing the formation of interlayer bridging bonds in the CNS. Click here for file

Additional file 4 **Bridged CNS**. A video showing the oscillation of a CNT housed inside an interlayer bridged CNS. Click here for file

## References

[B1] Viculis LM Mack JJ Kaner RB A chemical route to carbon nanoscrolls Science 2003 299 1361 10.1126/science.1078842 12610297

[B2] Shioyama H Akita T A new route to carbon nanotubes Carbon 2003 41 179 181 10.1016/S0008-6223(02)00278-6

[B3] Xie X Ju L Feng X Sun Y Zhou R Liu K Fan S Li Q Jiang K Controlled fabrication of high-quality carbon nanoscrolls from monolayer graphene Nano Lett 2009 9 2565 2570 10.1021/nl900677y 19499895

[B4] Braga SF Coluci VR Legoas SB Giro R Galvao DS Baughman RH Structure and dynamics of carbon nanoscrolls Nano Lett 2004 4 881 884 10.1021/nl0497272

[B5] Shi XH Pugno NM Gao HJ Tunable core size of carbon canoscrolls J Comput Theor Nanosci 2010 7 517 521 10.1166/jctn.2010.1387

[B6] Novoselov KS Geim AK Morozov SV Jiang D Zhang Y Dubonos SV Grigorieva IV Firsov AA Electric field effect in atomically thin carbon films Science 2004 306 666 669 10.1126/science.1102896 15499015

[B7] Zhang YB Tan YW Stormer HL Kim P Experimental observation of the quantum Hall effect and Berry's phase in graphene Nature 2005 438 201 204 10.1038/nature04235 16281031

[B8] Lee C Wei X Kysar JW Hone J Measurement of the elastic properties and intrinsic strength of monolayer graphene Science 2008 321 385 388 10.1126/science.1157996 18635798

[B9] Neto AHC Guinea F Peres NMR Novoselov KS Geim AK The electronic properties of graphene Rev Mod Phys 2009 81 109 154 10.1103/RevModPhys.81.109

[B10] Mpourmpakis G Tylianakis E Froudakis GE Carbon nanoscrolls: a promising material for hydrogen storage Nano Lett 2007 7 1893 1897 10.1021/nl070530u 17580924

[B11] Coluci VR Braga SF Baughman RH Galvao DS Prediction of the hydrogen storage capacity of carbon nanoscrolls Phys Rev B 2007 75 6

[B12] Shi XH Cheng Y Pugno NM Gao HJ Tunable water channels with carbon nanoscrolls Small 2010 6 739 744 10.1002/smll.200902286 20196087

[B13] Shi XH Pugno NM Cheng Y Gao HJ Gigahertz breathing oscillators based on carbon nanoscrolls Appl Phys Lett 2009 95 163113 163113 10.1063/1.3253423

[B14] Shi XH Cheng Y Pugno NM Gao HJ A translational nanoactuator based on carbon nanoscrolls on substrates Appl Phys Lett 2010 96 517 521

[B15] Zheng QS Jiang Q Multiwalled carbon nanotubes as gigahertz oscillators Phys Rev Lett 2002 88 045503 11801136 10.1103/PhysRevLett.88.045503

[B16] Legoas SB Coluci VR Braga SF Coura PZ Dantas SO Galvao DS Molecular-dynamics simulations of carbon nanotubes as gigahertz oscillators Phys Rev Lett 2003 90 055504 12633370 10.1103/PhysRevLett.90.055504

[B17] Rivera JL McCabe C Cummings PT Oscillatory behavior of double-walled nanotubes under extension: a simple nanoscale damped spring Nano Lett 2003 3 1001 1005 10.1021/nl034171o

[B18] Ershova OV Lozovik YE Popov AM Bubel ON Poklonskii NA Kislyakov EF Control of the motion of nanoelectromechanical systems based on carbon nanotubes Phys Solid State 2007 49 2010 2014 10.1134/S1063783407100332

[B19] Coluci VR Timoteo VS Galvao DS Thermophoretically driven carbon nanotube oscillators Appl Phys Lett 2009 95 253103 10.1063/1.3276546

[B20] Kang JW Song KO Hwang HJ Jiang Q Nanotube oscillator based on a short single-walled carbon nanotube bundle Nanotechnology 2006 17 2250 2258 10.1088/0957-4484/17/9/030

[B21] Zhang Z Li T Carbon nanotube initiated formation of carbon nanoscrolls Appl Phys Lett 2010 97 081909 10.1063/1.3479050

[B22] Kim YK Min DH Preparation of scrolled graphene oxides with multi-walled carbon nanotube templates Carbon 2010 48 4283 4288 10.1016/j.carbon.2010.07.039

[B23] Brenner DW Shenderova OA Harrison JA Stuart SJ Ni B Sinnott SB A second-generation reactive empirical bond order (REBO) potential energy expression for hydrocarbons J Phys: Condens Matter 2002 14 783 802 10.1088/0953-8984/14/4/312

[B24] Stuart SJ Tutein AB Harrison, J. A. A reactive potential for hydrocarbons with intermolecular interactions J Chem Phys 2000 112 6472 6486 10.1063/1.481208

[B25] Plimpton S Fast parallel algorithms for short-range molecular dynamics J Comput Phys 1995 117 1 19 10.1006/jcph.1995.1039

[B26] Coluci VR Legoas SB de Aguiar MAM Galvao DS Chaotic signature in the motion of coupled carbon nanotube oscillators Nanotechnology 2005 16 583 589 10.1088/0957-4484/16/4/041

[B27] Krasheninnikov AV Banhart F Engineering of nanostructured carbon materials with electron or ion beams Nat Mater 2007 6 723 733 10.1038/nmat1996 17906658

[B28] Kis A Csanyi G Salvetat JP Lee TN Couteau E Kulik AJ Benoit W Brugger J Forro L Reinforcement of single-walled carbon nanotube bundles by intertube bridging Nat Mater 2004 3 153 157 10.1038/nmat1076 14991016

[B29] da Silva AJR Fazzio A Antonelli A Bundling up carbon nanotubes through Wigner defects Nano Lett 2005 5 1045 1049 10.1021/nl050457c 15943440

[B30] Guo WL Guo YF Gao HJ Zheng QS Zhong WY Energy dissipation in gigahertz oscillators from multiwalled carbon nanotubes Phys Rev Lett 2003 91 125501 14525370 10.1103/PhysRevLett.91.125501

[B31] Xu ZP Energy dissipation in the double-walled carbon nanotube based mechanical oscillators J Comput Theor Nanosci 2008 5 655 658

[B32] Zhao Y Ma CC Chen GH Jiang Q Energy dissipation mechanisms in carbon nanotube oscillators Phys Rev Lett 2003 91 175504 14611358 10.1103/PhysRevLett.91.175504

[B33] Zhao XG Cummings PT Molecular dynamics study of carbon nanotube oscillators revisited J Chem Phys 2006 124 134705 10.1063/1.2185623 16613466

[B34] Kang JW Song KO Kwon OK Hwang HJ Carbon nanotube oscillator operated by thermal expansion of encapsulated gases Nanotechnology 2005 16 2670 2676 10.1088/0957-4484/16/11/034

